# Intraabdominal hypertension and abdominal compartment syndrome in patients admitted to a medical and surgical ICU at a third referral university hospital

**DOI:** 10.1186/cc12169

**Published:** 2013-03-19

**Authors:** N Chindavech, S Yenarkart, S Kongsayreepong

**Affiliations:** 1 Siriraj Hospital, Mahidol University, Bangkok, Thailand; 2 Division of Critical Care Medicine, Bangkok, Thailand

## Introduction

Intraabdominal hypertension (IAH), especially abdominal compartment syndrome (ACS), can affect organ function leading to multiple organ failure. Appropriate and prompt management could improve survival. Less recognition of this problem in critically ill patients has been reported. The aim of this study was to study the prevalence, predictive factors and clinical outcome of IAH and ACS in a mixed population of critically ill patients by intermittent measuring bladder pressure during the ICU stay.

## Methods

This prospective observational study was done in 130 adult patients (age >18 years) admitted to a medical and general surgical ICU at a third referral university hospital during June to November 2011. Variables about the patient's profile laboratory data and clinical outcome as ICU and hospital length of stay, and ICU, in-hospital and 28-day mortality were recorded.

## Results

There were 33 (25.4%) medical and 93 (74.6%) surgical ICU patients in this study. Surgical patients had higher prevalence of IAH than medical patients (57.8% vs. 33.3%, *P *= 0.015). Medical patients were admitted with severe sepsis/septic shock, AKI, pneumonia and ARDS. Surgical patients were more acutely ill with high ASA (III to IV), severity score, underwent emergency abdominal surgery and received more transfusion but were no different in type of fluid replacement. Significant risk factors of IAH were coagulopathy (OR = 2.09, 95% CI = 1.62 to 2.69), intraabdominal infection (OR = 1.87, 95% CI = 1.40 to 2.48), retroperitonium hematoma (OR = 1.82, 95% CI = 1.36 to 2.44), marked ascites (OR = 1.76, 95% CI = 1.32 to 2.36), acidosis (pH <7.2) (OR = 1.82, 95% CI = 1.37 to 2.43), severe sepsis/septic shock (OR = 1.63, 95% CI = 1.14 to 2.33), and massive transfusion (OR = 1.51, 95% CI = 1.10 to 2.08). Patients with IAH had more reopened surgeries and had higher ICU, hospital and 28-day mortality. Sixteen (12.3%) patients had ACS, 15 patients underwent emergency surgery, two patients had temporary abdominal closured and one-half of the patients had severe abdominal sepsis and massive transfusion. Fourteen patients died despite temporary abdominal closure. Delayed release abdominal tamponade were most causes of death.

## Conclusion

The prevalence and morbidity/mortality of IAH and ACS were high in this institute. Early appropriate and prompt management, especially fluid and releasing a tamponade effect, were important.

